# The impact of the first millennial teachers on education: views held by different generations of teachers

**DOI:** 10.1007/s10639-023-11768-8

**Published:** 2023-04-19

**Authors:** Juan José Marrero Galván, Miguel Ángel Negrín Medina, Abraham Bernárdez-Gómez, Antonio Portela Pruaño

**Affiliations:** 1 Department of Specific Didactics, University of La Laguna, La Laguna, 38200 Spain; 2 Department of Didactics and School Organization, University of Murcia, Murcia, 30100 Spain

**Keywords:** Teachers, Intergroup relations, Digital natives, Digital migrants

## Abstract

The first people considered digital natives, the millennials, have already entered the teaching profession. As a result, we are faced with a remarkable generational diversity. This survey aimed to explore the generational change in teachers and the beginning of the incorporation of the first millennials (digital natives) into teaching. It was carried out through a qualitative study using focus groups and interviews with a total of 147 teachers. The main results found establish a generational clash between migrants and digital natives. This difference is present in the use and understanding of ICTs in the teaching task across the different teaching generations and in a generational diversity within the educational centres that has not been seen so far. However, this difference between teachers is also a condition that facilitates exchange between teachers of different generations. Junior teachers help veteran teachers in the use of ICTs and veteran teachers provide the expertise that new recruits lack.

## Introduction

The COVID-19 pandemic dramatically increased the speed of social change. This has bewildered the population, with schools also being affected. Pandemic-induced closures meant that both schools and teachers had to ensure continuity of education provision and quickly respond to the new teaching and learning scenarios (Cabero-Almenara, 2020; Moreno-Rodríguez, 2020; Rodicio-García et al., 2020).

For Kuric et al. (2021), it would not have been possible to confront these new situations without Information and Communication Technology (ICT). ICT has shifted the traditional concept of literacy to a technology-based one, consistent with the current fluid and digitised society (Area, 2014; Pérez-Escoda & Pedrero, 2015). This is why ICT has had such a remarkable impact on education systems in recent decades. The adaptation of curricular materials and their migration to digital environments has brought about changes not only in teaching and learning processes, but also in the development of virtual learning communities and new forms of communication. ICT has changed the way schools and teachers communicate with students and families (Weisberger et al., 2021), leading to a more fluid educational model where traditional roles in the classroom may be altered (Chiang & Karjalainen, 2022).

In turn, the digital world is volatile, complex and ambiguous. It exerts a strong pressure on what is taught and why, calling for new curriculum designs (Elizondo, 2020) and learning environments (Gisbert & Lázaro, 2020) through the use of ICT. This makes digital literacy a key objective for the education system (George, 2020). For this objective to be met, critical thinking needs to be part of the process, in order to ensure security and protect the digital identity of learners; enable them to select information through critical reading; collaborate in digital spaces; know how to make intelligent use of the available information; and transform that information for use in virtual scenarios (George, 2018).

However, teachers will not be able to use ICT to teach these digital literacy skills unless they have previously acquired an advanced level of digital skills themselves (Cabero, 2014; González et al., 2012; Llorente, 2008). The study carried out by Pérez and Rodríguez (2016) showed that teachers had a poor self-perception of their digital skills with respect to indicators such as digitally storing information, collaborating in teams by using digital channels, sharing materials through online tools, editing and producing materials, having computer skills, using proprietary rights or choosing appropriate software.

The COVID-19 pandemic has dramatically highlighted this shortfall in teachers’ digital skills (Cabero-Almenara, 2020), which, according to Cabrera (2020), could be related to generational differences between teachers. For Köttl et al. (2021), teachers perceive older generations as lacking the ability to use digital technologies, and attributed this to the decline in physical, social and personal skills associated with ageing. However, it is interesting to note that the pandemic also stressed the disparity in the level of digital skills among teachers; during the lockdown period, teachers with better digital skills were able to share their skills and knowledge of how the online learning platforms used during the period operated with those with weaker skills (Boix, 2020).

Does this mean that the new generations of teachers have acquired sufficient skills based on the technological development of ICT? According to Prensky (2001a, 2001b) and Tapscott (2009), these teachers should belong to the group of so-called digital natives (born between 1980 and 1994). This group is characterised by being technophiles and having sufficient computer skills and fully confident in the use of ICT, as they have grown up in a fully digital environment (Prensky, 2001a, b). In contrast, so-called digital immigrants come from an eminently analogue background and have learnt to use ICT to adapt to an increasingly technological society (Rodicio-García et al., 2020).

Numerous authors have questioned this classification by arguing that it is based solely on age or generational criteria (Bennett et al., 2008; Cerezo, 2016; Creighton, 2018; Gros et al., 2012; Kennedy et al., 2010; Lenhart et al., 2008). For Kirschner & De Bruyckere (2017), digital natives do not necessarily have full command of ICT. Rather, digital natives must have the ability to cognitively process multiple sources of information simultaneously (multi-tasking), which means that they need a different educational approach to that of previous generations (Kirschner & De Bruyckere, 2017).

According to Prensky (2001a, 2001b) and Tapscott (2009), while millennials (those born between 1981 and 1996, as defined by Dimock, 2019) are considered to be digital natives, baby boomers (those born between 1965 and 1990, as defined by Dimock, 2019) are regarded as the generation of digital immigrants. For Cerezo (2016), this simple classification is unsatisfactory, since it implies that all members of the millennial generation are technologically trained. This would make millennials a generation of ‘digital integrators’ (McCrindle, 2015), or one that has acquired ‘digital wisdom’ (Prensky, 2009), which is linked to their attitude towards ICT and educational and training factors, but not to age or social position. Regarding baby boomers, McCrindle (2015) uses the term ‘digital transactors’ to describe those who are able to functionally use ICT to perform tasks that were previously completed through analogue technologies.

In addition, despite the digital divide between baby boomers and millennials, both groups have shown a willingness to be trained in certain digital skills that are adapted to the requirements of each generation (Jiang et al., 2016). A significant group of in-service teachers (aged between 40 and 60) are digital immigrants who have adapted to ICT in order to understand learners defined as digital natives, and address their educational needs through the use of these technologies (Creighton, 2018; Romero-López et al., 2022). For Salas (2020), it is only possible to educate digital natives (such as millennials) if there are synergies between the skills of these students and those of their digital immigrant teachers (mainly baby boomers). This requires a positive attitude on the part of these two generations regarding shared learning in the use and application of ICT.

Baby boomers are currently and gradually being replaced by millennial students and teachers in the classroom. Both groups of millennials need to have their creative demands met in learning and teaching processes, driven by the unlimited technological resources available (Montes de Oca, 2017). The decline in the number of baby boomer teachers compared to new millennial teachers seems to have accelerated as a result of COVID-19. This is due to the fact that the former had considerable difficulties in adapting to the new digital educational environment, which require teachers to have strong technological, communication and teamwork skills in order to improve the teaching and learning processes for online education (Vargas-Rodríguez et al., 2021).

The research project entitled ‘Intergenerational Professional Development in Education: Implications for Initial Teacher Training (known by its abbreviated form in Spanish as ‘DePrInEd’)’ (Portela et al., 2022) aims to generationally characterise teachers and the interactions between them during their professional practice. The use of ICT in the classroom, including its technological evolution and the changes it has brought to teaching, seems to be one of the processes that has had the greatest impact on the different generations of teachers during their professional development. The confluence of baby boomers, digital immigrants and the new millennial, digital native teachers in schools could provide a new perspective on how ICT could mark the generational replacement of teachers, accelerated by the COVID-19 pandemic in Spain.

Some questions therefore arise in the face of this generational confluence: What has been the impact of the first millennials joining the teaching profession? How have digital immigrants and millennials themselves experienced it, and what relationships have emerged between them?

## Study focus and design

A qualitative methodology was used in this study, as it gave researchers a holistic, in-depth understanding of participants’ experiences in real-world contexts, and gives access to what these experiences mean to them (Denzin & Lincoln, 2018). This methodology was used by adopting a constructivist-interpretivist approach. According to this approach, participants construct meanings in interaction with their context. The researcher can uncover the subjective meanings that participants give to their experiences by interacting with them (Denzin & Lincoln, 2018; Merriam & Tisdell, 2016).

This research was designed as a basic qualitative study (Merriam & Tisdell, 2016). It was also a comparative, cross-sectional (or snapshot) study, as the researchers’ focus was on comparing individuals and groups at a single point in time (Flick, 2018). As in other qualitative studies (Creswell & Poth, 2018), an additional main feature of the study was that it used a sequential combination of methods, namely, focus groups and interviews. The former were first used to explore the under-researched aspects of interest by drawing on a range of views (Hennink, 2014), and the latter were next used to examine them in a more comprehensive, detailed manner from a more personal perspective (Patton, 2015). This design was used to gain information about the different aspects of the phenomena of study, and thus acquire a greater depth of understanding, but also to triangulate different methods (that is, different methods were used as a check on each other) for validation purposes (Maxwell, 2013).

### Participants, sampling, and recruitment

The participants (n = 147) were all teachers in state-funded schools. For their selection, two criteria were applied to differentiate and group them by generation: age and teaching experience. This combination of criteria has been used in similar research (Flick, 2018). In addition to teachers currently in employment, some retired teachers were included as participants because it has been shown that there is continuity and even further development of their professional identity as teachers beyond retirement (Shlomo & Oplatka, 2020). The full set of inclusion criteria are listed in Table 1.

**Table 1:** Inclusion criteria

	Young Beginning Teachers (YBT)	Mature Veteran Teachers (MVT)	Older Retired Teachers (ORT)
Age and teaching experience	Aged 30 and younger (born after 1990) with teaching experience of 6 school years or less.	Aged 50 and over and teaching experience of 10 school years or more.	Aged 60 and over (either by compulsory retirement or by voluntary retirement).
School levels taught	Infant Education (2nd level), Primary education Secondary education		
Type of school	Publicly-funded schools		

Participant selection used purposeful sampling, in which choice is based on the wealth of information they can provide (Emmel, 2013; Patton, 2015). A combined sampling strategy was used to study homogeneous groups and examine the maximum variation within them to document their diversity and identify common aspects (Patton, 2015). A national teachers’ union and several retired teachers’ associations were involved in the recruitment of participants. Researchers’ personal networks and social media were used to complete the sample. The final number of subjects in each category was in line with the sample sizes suggested in the literature and those necessary to reach a sufficient level of saturation (Hennink & Kaiser, 2022; Sim et al., 2018).

The characteristics of the participants can be found in Table 2. This information was provided by means of an electronic questionnaire in which they gave their informed consent. The information was cross-checked when contact for data collection was initiated.

**Table 2:** *Characteristics of participants*

	YBT (n = 51)				MVT (n = 50)				ORT (n = 46)			
	Mean		SD		Mean		SD		Mean		SD	
Age^a^	28		1.9		56.6		2.9		66.9		4.6	
Teaching Experience^b^	2.8		2		27.6		6.5		35.7		6.2	
	YBT			MVT			ORT			Full sample		
	n	%		n		%	N	%		N		%
School stage												
Infant/Primary ^c^	27	52.9		24		48	23	50		74		50.3
Secondary	24	47.1		26		52	23	50		73		49.7
Gender												
Female	37	72.5		38		76	23	50		98		66.7
Male	14	27.5		12		24	23	50		49		33.3

^a^Age ( on 31 December 2021.

^b^Years’ experience when answering the initial electronic questionnaire.

^c^This category includes early childhood education (2nd stage) and primary education.

### Data collection

Data collection was carried out through focus groups followed by interviews. The focus groups were conducted with a larger number of participants (n = 24 × 6 participants). They were conducted through exchanges and discussions among participants on topics of interest to the participants. In this way, information emerged from the interaction of various points of view (Hennink, 2014; Stewart & Shamdasani, 2015). The focus groups were used for exploratory purposes. Interviewees (n = 60) were selected from among focus group participants to be able to delve further into issues of interest and provide more detailed insights (Onwuegbuzie & Collins, 2007; Patton, 2015).

**Fig. 1:**
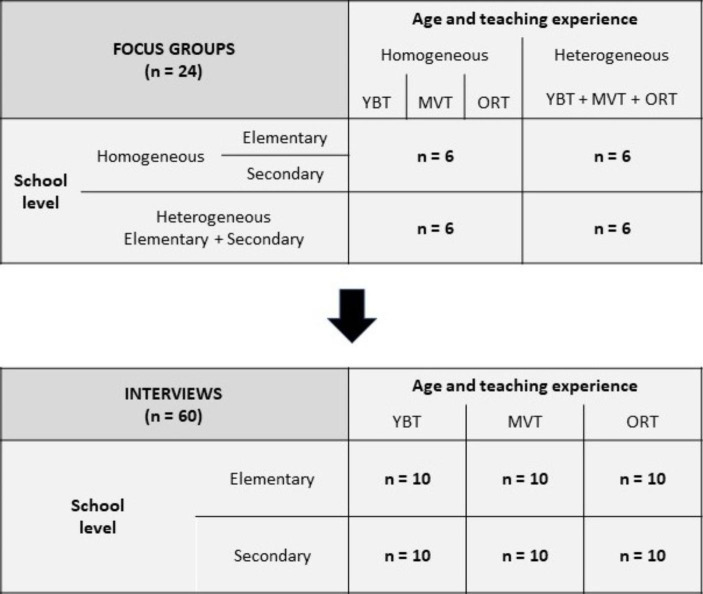
*Segmentation of focus groups and interviews*

Both data collection techniques were segmented (Hennink, 2014). Two characteristics of the participants were taken into account: teaching experience and school stage taught. A balance was sought between the different criteria used. In this way, the aim was to access both similar and different points of view at the same time. Figure 1 shows the segmentation carried out.

Both data collection techniques (focus groups and interviews) were semi-structured. The protocols and guidelines developed by the research group were used, tested and improved. In both cases, core questions and a series of probing and follow-up questions were used if the initial response was poor (Patton, 2015). The topics addressed in the core questions referred to the participants’ view of the teaching profession and their professional history, which generations they could identify among the teaching staff, and what characterises them, how they have experienced their own teaching over time and with whom they share their view of the profession. Participants were encouraged to focus on their motivations to become teachers, including intrinsic motivation.

Data were collected via videoconferencing due to the different constraints imposed by the various pandemic waves and the characteristics of the participants (given the advanced age of some of the respondents). The focus groups were conducted by one or two members of the research team and the interviews were performed by one researcher. The duration of the focus group sessions ranged from 61 to 164 min, with an average duration of 93.4 min. The length of the interviews ranged from 28 to 136 min, the average length being 66.3 min. They took place between April and December 2021.

### Data analysis

After obtaining permission from the participants, the focus groups and interviews were audio-recorded and fully transcribed verbatim. A reflexive analysis was conducted on the transcripts, placing special importance on researchers’ critical reflection on their own assumptions and decisions. Reflexive thematic analysis was used, following the steps outlined by Braun & Clarke (2012, 2022) and Terry & Hayfield (2021). This was combined with the constant comparative analysis method (Leech & Onwuegbuzie, 2008), which involved a recurrent comparison of data units in order to enhance the analysis. These two methods provide flexibility in the analysis, which was the main reason for selecting them.

Drawing on the method described by Braun and Clarke (2012, 2022), the data analysis was carried out over a series of stages or phases that were, in turn, sequential and recursive. This characteristic of the analysis meant that the different phases could overlap, given the reflective process inherent in the model used. The following phases were used:

- Familiarisation with the data by the team, through an analytical and focused reading of the content.
- Codebook development, which involved segmenting meaningful data to identify, define and exemplify codes. A balance was struck between data-driven analysis and theory-driven analysis (Braun & Clarke, 2012). The coding was carried out by the members of the research group, who held meetings to discuss and reflect on the coding done so far.
- Generating initial themes, a phase that entailed looking for themes to identify patterns that related the dataset to the research questions, using core categories organised in different code clusters with shared features.
- Reviewing themes to check their quality by comparing them with the data obtained in order to confirm, correct or reject the core categories.
- Final naming of themes in order to refine them and linked them to others; this also involved generating definitions and collating them with relevant data extracts related to the situation described to help clarify the theme.

The qualitative analysis was carried out using ATLAS.ti V22 (Scientific Software Development GmbH) because, according to Soratto et al. (2020), it makes it possible to easily display qualitative information of interest that may be underlying the figures that provide a compare-and-contrast dynamic between the codes or categories assigned to the dataset.

### Ethics

Participants received detailed and accessible information about the study and the conditions of participation, including their right to revoke their voluntary participation and guarantee of confidentiality and anonymity. Participants were asked to provide their informed consent, which they gave individually in writing via a short electronic form. This consent was confirmed orally upon their participation in focus groups and interviews. The project of which the study forms part has been reviewed and approved by the Ethics Committee of the University of Murcia (Approval identification code: 2087/2018).

## Results

After completing the analytical process, a category emerged in connection with the context where teachers work that referred to the role of ICT in the school, its expansion and its widespread penetration in educational settings. This category of interest for the analysis or core category was given code 1.09_About_Context: 1.09.12_Scope of ICT. When this particular code was analysed, it was noted that there was a constant reference to change, innovation or updating in teachers, which in turn generated another central category that would be of interest in our analysis: 1.02_About_Teachers: 1.02.11_Change. Having confirmed the relationship between them using the co-occurrence tool in ATLAS.ti, it was decided to further investigate the relationship between change in teachers and the breadth and scope of new technologies. A code emerged from the data that had not been considered until now. Code 1.02_About_Teachers: 1.02.17_Digital natives covered data extracts referred to millennials who had entered the teaching profession and were considered digital natives by their older colleagues. The following results were shaped around a series of specific issues of interest: generational change in teachers and influx of first millennials into teaching for the first time; the characterisation of digital native teachers; and the impact of their entry into an ecosystem dominated by digital immigrants.

### Teachers as subjects of change

Throughout their career, teachers engage in a considerable number of processes that bring change into their work. As they go through these processes, teachers must evolve with the requirements of society and their students. However, the process related to the introduction of technologies has not been a sporadic occurrence. On the contrary, the implementation of new ICT resources or methodologies in schools is a constant which does not seem to be coming to an end any time soon. As a result, teachers are continuously evolving with their students:

students handle all of these technologies. In fact, they teach me lots of things every day… When I go to the classroom, I start to explain something to them…, ‘Hey, Pepa, you can do it better this way’ and they immediately help me, and together we do it better. (MVT-FG [Focus Group])

**Fig. 2:**
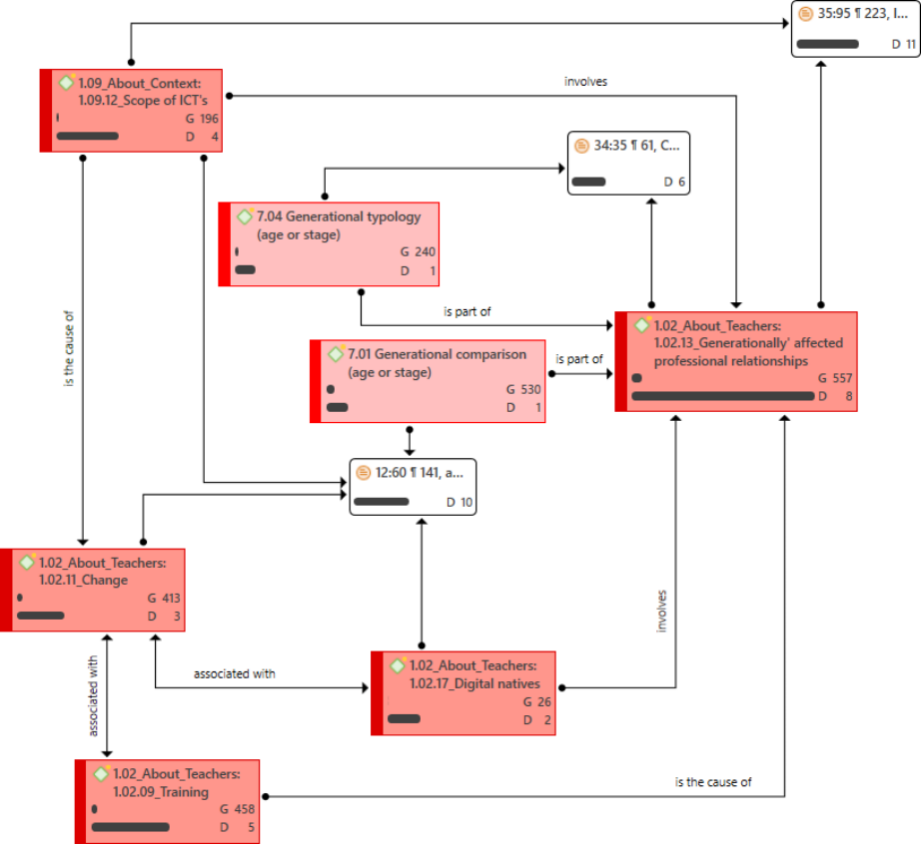
Semantic network of relationships between codes associated with ICT-related change in teachers.^1^

Teachers see very clearly the change in their profession as brought about by new technologies (Fig. 2). This change is succinctly related to other changes facing teachers, for example, lifelong learning to keep up with the frantic pace of technological developments. However, one of the main implications of new technologies is that generation-based professional relationships are affected by these developments. As will be seen in the following sections, digital natives who have become teachers, and have grown up with technology, are a new type of teacher. This gives rise to comparisons in terms of the interaction that teachers of different ages have with ICT.

For the sake of clarity, each of the categories related to the topics of interest will be explained. This will be followed by a description of the categories. Table 3 provides a brief explanation of what the researchers understood each of these codes to mean.

**Table 3:** *Related categories that emerged from the analysis*

Categories related to the core ones	
Change	The participant highlighted change, and even innovation or updated skills among teachers (including readiness to change or update one’s skills) or lack of change or readiness to update one’s skills.
Training	The participant highlighted a teacher’s preparation or training (including their readiness to engage in preparation or training programmes) or lack thereof.
Digital natives	The teacher who was discussed in the data extract is considered a digital native.
Impact of generational factors on inter-generational relationships	The participant highlighted the professional and/or other relationships between professionals (including collaborative ones) or the shortfalls that were affected by the generational affiliation of the teachers in question belong.

### Change in teachers

The change brought about by ICT is an issue that appeared at an early stage in the analysis. Most of the participants mentioned aspects such as increased work levels, in other words, the added burden that technology has placed on them, particularly on older teachers, ‘teachers in my generation, who are more or less the same age as me, largely find new technology overwhelming’ (ORT-IN [Interview]). However, more veteran teachers perceived that this was not the case for younger teachers. Many of the newcomers belong to a generation in which the introduction of ICT has brought a change with respect to their predecessors, ‘more new teachers are coming in, and with the technology boom… I don’t know, a change has occurred that can also be noticed in our closeness to students’ (YBT-IN).

Most of the participating teachers agreed that the main changes are gradually being introduced in education, as they take place in society. However, they also referred to the influence of certain events as motivators for change and the introduction of ICT. The changes resulting from the COVID-19 pandemic were also prominently featured in their discourses. This has been one of the main reasons for the further introduction of ICT in schools. One of the new teachers pointed out: ‘in two years we have been forced to learn very, very fast’ (YBT-FG). A veteran teacher meanwhile highlighted the difference in how they teach, and the forced change and adaptation brought about by social need during the pandemic period:

We had rudimentary, domestic knowledge of a few things, but the sudden change that the pandemic meant for me… the fact that I had to teach online from home, because I had students in the second year baccalaureate who had to take the… for me, it was… (apart from being a burden), a very big worry. It meant getting down to work and training myself the best I could at home, with basic resources. (MVT-FG)

According to a retired teacher who did not experience the pandemic in the classroom, society is changing and teachers must adapt; otherwise they would be doing their job the wrong way: ‘technology has changed the world, it is changing the world, it is changing from early childhood, so to consider whether… to use it or not to use it with my old teaching methods is to make a complete mistake’ (ORT-FG). Consequently, this change involves an added difficulty and a need that did not exist before. This process entails an increased workload and the challenge of adapting to ICT. The need for training to overcome this challenge grows with time and the introduction of new tools, and there has been an attempt to minimise the difficulty through ongoing teacher training. This is intended to reduce any negative impact of technology and to meet students’ needs. The veteran and retired teachers who most vehemently emphasised the need for ICT training argued that this should be an ongoing process, ‘and you have to keep updating your skills, but it’s not that easy but you try and… say well then, it looks like I’m going to do such and such and you get on with it and go ahead’ (MVT-IN). This is a burden borne by these teachers. However, new teachers already have an ICT knowledge base, and therefore they do not require as much training to use these tools. This is how veteran teachers perceive novice teachers:

I think that the new people who are coming in right now, who recently passed their competitive examinations to start teaching, have new methods that I find interesting, and they have learnt this. But when I did my training, of course these technologies didn’t exist, but it seems that young teachers have been taught specific methods that are closely related to ICT and are given ICT training (MVT-IN).

As noted by the older teachers who participated in the study, their novice colleagues are the first generation of teachers who can be considered digital natives. These teachers come from generation Y, the so-called millennials, who are digital natives rather than digital immigrants. There was therefore a view among the participants that there is a gap between the digital natives and their predecessors. The next section analyses the observations made about these teachers to assess whether there is in fact a gap between the generations.

### Implications for the first digital native teachers

The previous sections showed the differences found by the participants regarding the use and knowledge of technologies according to age and stage in their career. Novice teachers have an instinctive grasp of technology, as they had ICT training at school, and they have always used these tools at home as well. These younger teachers saw the implementation of the resources that are now available in their student days. One of them said: ‘when I was taught in secondary school, for example, there was already a bit of an ICT hype, you know. Everyone was on their mobile phones, there were computers, tablets, all those things too’ (YBT-IN).

Having grown up with very similar technological tools to those currently used with their students, they perceive these tools differently than their colleagues. Previously, older professionals pointed out their difficulty in using ICT in their daily lives, which is not the case for young people, this is normal for them and something that they are competent in. Young teachers have internalised technology in such a way that they do not see it as an issue that they need to consider on an ongoing basis. Thus, the older teachers noted that novice teachers do not have the same concern as them about the introduction of ICT: ‘I think they don’t see it as a serious issue, they have a high command of technology’ (MVT-IN).

The ease with which they use ICTs has a positive impact on their daily work, including building a rapport with and reaching out to their students. As reported by older teachers, students think that novice teachers understand their needs better. Young teachers grew up with similar technology as their students, and have a greater ability to relate to learners. A retired teacher stated: ‘children already live in the digital world—they have done since they were little—and they have a better rapport with these young teachers because they have a high command of technology, unlike us… it’s harder for us’ (ORT-IN).

Furthermore, in addition to the reported rapport of younger teachers with students, another type of rapport was also highlighted between the more technologically savvy teachers. Despite the challenges that technology poses and its benefits for teachers who instinctively use it in their work, there are some social problems which are then transferred to the teaching environment. This habit of being constantly online was seen by many older or retired teachers as harmful. A veteran teacher mentioned: ‘I have colleagues who hold their mobile phones in their hands all day’ (MVT-FG). This may result in dependence from technology such as social networks or information overload. A retired teacher noted that ‘it is a generation that is becoming intoxicated by so much information. They are intoxicated, as they are receiving information through Instagram, Facebook, this, that, everyone is on WhatsApp, and so on’ (ORT-IN).

The different points and subthemes within these themes may give rise to differences which have not been strongly challenged to date. The digital divide between different generations of teachers is therefore a specific issue that may be triggered by the use of technologies.

### Technology-based comparison between the generations of digital natives and digital immigrants, millennials vs. boomers

The extent of the digital divide among teachers was one of the issues most frequently mentioned by the teachers who participated in the study. They talked about a generation gap being created as a result of the use of technology. ICT is one of the reasons why some teachers felt displaced. Just as water gradually erodes the soil through which it flows, sweeping changes such as those brought about by technology have a similar effect on teachers who, for a variety of reasons, are unable to keep up with these developments in their profession. ‘The new technologies also have an impact and, whether you like it or not, those of us who are already in this age group are being pushed to one side or are lagging behind in this new trend…’ (MVT-IN). However, this view was not only expressed by the older teachers, as it was also reflected in the discourse of some of the younger teachers, even though in the form of a positive comment on their more experienced colleagues.

Right now I have a colleague who is an ‘older woman’ (she is 54 or 55 years old), and she is constantly asking me for help: ‘Look, that stuff that you were telling me about the other day, can you…?’. This woman is eager to learn, she is eager to put into practice the things she hears about and to innovate. (YBT-FG)

The most important implications and consequences identified were those related to the digital skills and competences that each of them has: ‘In the field of technology, basically, it’s what they use every day and well, you’ve seen how hard it was for me to get a connection; we’re not as skilled as these people are’ (ORT-FG). One of the main differences that participants reported was the methodologies used by the various generations of teachers. Older teachers follow more traditional trends in their work because they do not use ICT:

… those in my generation don’t update the content they teach very much; […] those who have been working for about five years, who are younger, between twenty and thirty years old, have a slightly different vision, perhaps a little more open to more innovative learning methods, more didactic, they use ICT much more. (ORT-IN)

Occasionally, older teachers see the natural, highly skilled technology literacy of their younger colleagues as a threat. This highlights a degree of insecurity that this phenomenon causes as it changes the way they work and is often difficult to manage. A veteran teacher stated:

the last two years, when a wave of very young people came in, and sometimes they were a bit too excited about technology, using Genially, podcasts, I don’t know, lots of other stuff, come on, come on… I haven’t used those tools, but if you show me how it works, I’ll get to work, because I was one of the first people to get involved in everything, in this computers and technology business, among other things, because I was on the management team and the position also required me to handle a lot of technological things. (MVT-FG)

These issues bring to the fore the digital divide between teachers of different generations. There is clearly a gap in the tools the different generation types use in their teaching, that is, between teachers who are digital natives and those who are digital immigrants. A veteran teacher indicated: ‘when I started teaching all it really involved was a tape and a book, a method with a tape, then the computer was introduced and you had your diskette with the method (your *diskette* with the method)’ (MVT-IN). Veteran teachers, who once experienced the introduction of educational technologies in the classroom using analogue methods, are now immersed in a new technological revolution that has been more difficult for them to keep up with.

It is true that we have had to adapt to many dramatic changes, that we are purely and simply the outcome of a huge transformation, because we were born without technology. When we started working we used Freinet’s printing press, we used it to make photocopies, and now we send stuff through the cloud, right? But I see all this as a personal challenge (ORT-FG).

However, in the relationships between teachers, the differences caused by technology were not only seen as being negative. Young teachers, as digital natives, were aware that they needed the experience of their older colleagues. They were aware that a greater knowledge of technology is not enough to deliver an excellent teaching performance, and they sought the help of senior teachers to solve problems they had not yet experienced.

We have just started in this (career) and very similar things happen to us. We are not[…] there are some situations that we haven’t encountered yet and are new to us, and maybe if we discuss them we realise that the same thing that happened to us occurred to our older colleagues. The other, more experienced generation is already familiar with these things (YBT-IN).

In these cases, there is a trade-off between what is most valuable to each group of teachers: experience for one group and technological skills, for the other.

I believe that the new generations contribute a lot of technology, a lot of academic knowledge as well, and the older generations bring experience, and that experience has depth, it has serenity, it has calmness, and it is very good. It has brought me a lot. (ORT-FG)

Therefore, an exchange occurs between teachers of different generations. Older professionals are a source of experience for novices who are unfamiliar with some basic notions of teaching practice. In turn, young people facilitate the use of ICT in the school setting. A veteran teacher pointed out that ‘thanks to new people, they basically opened doors for those of us who were inexperienced in technology and facilitated our access to this world, which is really wonderful’ (MVT-FG). Another veteran teacher added that digital native teachers are an incentive that stimulates them in their learning of and immersion in ICT:

Well, having a young person who makes you feel like ‘ok, I’m going to get my act together with this stuff now’. Otherwise… they’re like - ’if she doesn’t do it, I’ll do it myself…’ but then they have to be the actual ‘doers’ because I tell you, technology drives me mad, it drives me mad because it’s very difficult for me. (MVT-FG)

This reciprocal relationship was reported by most of the teachers as a positive exchange, since they valued new teachers’ ICT ability and knowledge, ‘I think this is a positive thing, digitalisation or the use of digital technologies by young teachers, who already use them as I used to use chalk’ (ORT-IN). This also results in young teachers’ enthusiasm for ICT, as noted above. These novice teachers, who are so skilled in new technology, are the most intent on its application or possibly the most aware of the need for ICT to be used.

… differences the most important thing is technology […] I think that I (or my generation) place more importance on using tools and teaching them and handling them and so on, than older teachers do… I’m not saying that they don’t find them important, it’s more that our generation has had more experience of them (YBT-IN).

However, older teachers ended up perceiving these differences as being very positive, as it ensured that this new generation of teachers can adapt to the experiences currently faced by students and build a good rapport with them.

## Discussion and conclusions

The main goal of this study was to explore the generational change in teachers and the influx of millennials (digital natives) into the teaching profession for the first time. The characteristics of digital native teachers and the impact of their entry in schools, where digital immigrant teachers predominate, has been described and discussed. The teaching profession has certainly changed and become more demanding as a result of the inclusion of technology in teaching (Weisberger et al., 2021), and also because there is often a mismatch between practice and implementation (Azaza et al., 2021).

The results presented here have clarified the answers to the research questions initially posed. Regarding the first question (What has been the impact of the first millennials joining the teaching profession?), we have been able to observe that the teaching profession has undergone considerable changes due to the emergence of ICT. This technology has proven to be significantly challenging for teachers, at least in the initial stages. However, with the entry of the first ICT-trained teachers into the profession, they have become allies to digital immigrant teachers in their use of these complex tools. This leads to the second question (How have digital immigrants and millennials themselves experienced it, and what relationships have emerged between them?). What a priori could be seen as an element that may hinder the relationship between teachers, has turned out to be the opposite: a great facilitator of intergenerational relationships. The digital divide has galvanised teachers into joint action to enhance their daily efforts. As a result, the relationships between them have been even further strengthened .

Changes in education mirror those that occur in society, although certain events or triggers for change also play a role. Thus, for example, the outbreak of the COVID-19 pandemic accelerated technological change, an aspect already mentioned in other studies (Montenegro et al., 2020; Williamson & Hogan, 2020). This change has caused additional difficulties for teachers that did not exist before (increased workload, adaptation to technology, new methodologies, etc.) and an imperative need for ongoing training. As noted by Aguiar et al. (2019), the pace of ICT implementation is hindered by traditional teaching and learning models. The veteran teachers interviewed believed that new teachers who join schools today (millennials) are digital natives who have grown up with technology, have a good technological knowledge base and do not need as much training to work with these tools; in short, they are better adapted to the changes that are taking place. The differences identified by the participating teachers in terms of the use and knowledge of technologies according to age or career stage were repeatedly mentioned, which suggests that generational identity plays an important role in the way teachers view educational change (Stone-Johnson, 2014).

Regarding teachers’ understanding of professionalism, Stone-Johnson (2014) pointed out that not only has it changed over time, but groups of teachers experience and understand professionalism differently. Thus, teachers in the boomer generation have different perspectives and therefore deal with the changes taking place in schools differently. Similarly, it is to be expected that millennial teachers who are technologically savvy do not have the same view of the teaching profession. Having grown up with similar technological tools to those they must now use with their students means that they perceive their use differently than some of their colleagues.

It is important to highlight three aspects that veteran teachers reported concerning how they saw millennial teachers: their integration of technology in their teaching, their closeness to students and their being constantly online. Regarding the integration of technology, the mere use of ICT is not bringing the improvement that was initially predicted (Cuban, 2008; De Pablos et al., 2010), and one of the main causes of this situation is that teachers do not have the necessary basic ICT knowledge and skills (González & De Pablos, 2015). There is also a notable difference between the skills that teachers should possess to inculcate digital competencies in students and those they actually have (Falcó, 2017; Han & Nam, 2021; Padilla, 2018). However, in the view of senior teachers, this is not the case for young teachers. Likewise, the ease with which young teachers use ICT has a positive impact on their daily work, especially in terms of developing a rapport with, and getting closer to, their students, as they have a better understanding of their needs. For example, the influence of social networks and connections, as well as Generation Alpha’s high ability to interpret information, are to be considered when developing future approaches to the teaching-learning process (Ziatdinov & Cilliers, 2021). In principle, millennial teachers are instinctively prepared for this, resulting in a good interpersonal relationship with their students, which is key in the educational process (Beaudoin, 2013). Finally, the third aspect to which they referred has a negative connotation and refers to the fact that young teachers have problems that are specific to the social sphere, which are consequently transferred to the teaching sphere. A way of life where the individual is constantly connected to technology is seen by many older teachers as being detrimental.

The results obtained also reflect the importance of the digital divide among teachers, facilitated by excessive technification of society and schools, which is why veteran teachers feel displaced. This is in line with the observations made by Pérez and Rodríguez (2016) concerning older teachers having a negative self-perception of their digital competences. As shown by the methodology used by teachers and their teaching styles, older teachers follow more traditional trends because they do not use ICT and have difficulties in adapting to their students’ experience of it (Tafonao et al., 2020). This was even more pronounced during the coronavirus pandemic, when the wider digital divide (teachers, families, students) was uncovered (Fernández-Río et al., 2022). Ultimately, changes are taking place that affect the social structures of schools.

However, the differentiation generated by technology is not only negatively perceived by the various generations of teachers. Novice teachers (digital natives) are aware that they need to learn from the experience of their older colleagues. Meanwhile, older teachers see their younger colleagues as an encouraging stimulus for their learning and immersion in ICT; they see the differences in a very positive light in terms of what truly matters, that is, ensuring that this is a generation that is able to adapt to students’ current needs and build a rapport with them. Each of these groups of teachers contribute what is most valuable to them by exchanging experience and technological knowledge, respectively. Several authors (Bair, 2017; Nelsen, 2015) have noted that teachers’ dispositions change, and even develop, as they interact with their context. In addition, as Melasalmi & Husu (2019) noted, depending on a set of circumstances, even professional dispositions develop collectively.

The study shows that the impact of the entry of digital natives into the teaching career is related to the change from an analogue to digital society, and that the change in teachers is determined by this change, as is the gap that can exist between digital natives, digital immigrants, and those who refuse to change. The COVID-19 pandemic accelerated this divide, as different generations of teachers have different rates of adaptation to, and learning of, the use of technology to maintain an operational education system. In the face of this, veteran teachers seem to perceive digital natives as being technologically dependent and intoxicated by information overload through their use of social networks. This perception could be associated with the fact that many veteran teachers have not fully come to terms with their digital competence shortfalls; in some cases they find it threatening that digital teachers naturally use technologies that bring them closer to students than their traditional teaching style. As a final conclusion, it is encouraging that the different generations of teachers all noted the need for synergic processes to be developed between the experience of veteran teachers (digital immigrants or not) and the contribution of digital natives to the use of technologies and digitalisation in the educational process, as a normalised way for the generational changeover of teachers in the classroom.

These findings complement other studies on millennial teachers (Clark & Byrnes, 2015; Mäkinen et al., 2018; Tang et al., 2020), who are a key group to be studied because they will contribute significantly to the present and future of education. As this study has focused solely on the perceptions of teachers, future research could broaden the scope to collect data from other agents involved in teaching, especially students and their families.

## Data Availability

The data that support the findings of this study are available from Ministry of Science and Innovation but restrictions apply to the availability of these data, which were used under license for the current study, and so are not publicly available. Data are however available from the authors upon reasonable request and with permission of Ministry of Science and Innovation.

## Footnotes

1 Different elements can be observed in the network. The different themes analysed are in bright red. The codes referring to generational typology or classification are presented in a more subdued colour. Each of these codes are categories (central or related to our analysis) that are intertwined within a specific relationship that makes explicit the nature of the relationship between these categories. In addition, as the software does not allow the co-occurrence coefficient between two codes to be displayed to show the relationship, it was decided to display extracts that are representative of this relationship. Finally, the frequency of use (G) and the density (D), that is, the relationship with other codes is shown for each of the codes. In the case of representative extracts, the document (D) where they are located is shown.
